# Discrepancy in Response of Mouse Dendritic Cells against BCG: Weak Immune Effects of Plasmacytoid Dendritic Cells Compared to Classical Dendritic Cells despite the Uptake of Bacilli

**DOI:** 10.3390/tropicalmed8030140

**Published:** 2023-02-25

**Authors:** Chuang Meng, Jun Liu, Xilong Kang, Zhengzhong Xu, Shuangyuan Xu, Xin Li, Zhiming Pan, Xiang Chen, Xinan Jiao

**Affiliations:** 1 Jiangsu Key Laboratory of Zoonosis, Yangzhou University, Yangzhou 225009, China; 2 Key Laboratory of Prevention and Control of Biological Hazard Factors (Animal Origin) for Agrifood Safety and Quality, Ministry of Agriculture and Rural Affairs, Yangzhou University, Yangzhou 225009, China; 3 Jiangsu Co-Innovation Center for Prevention and Control of Important Animal Infectious Diseases and Zoonoses, Yangzhou University, Yangzhou 225009, China

**Keywords:** BCG, plasmacytoid dendritic cells, classic dendritic cells, immune response, in vivo

## Abstract

Tuberculosis (TB), a zoonosis characterized by chronic respiratory infections, is mainly caused by *Mycobacterium tuberculosis* and is associated with one of the heaviest disease burdens in the world. Dendritic cells (DCs) play a key role and act as a bridge between innate and adaptive immune responses against TB. DCs are divided into distinct subsets. Currently, the response of DCs to mycobacterial infections is poorly understood. Herein, we aimed to evaluate the responses of splenic conventional DCs (cDC) and plasmacytoid DCs (pDC), subsets to Bacillus Calmette–Guérin (BCG) infection in mice. Splenic pDC had a significantly higher infection rate and intracellular bacterial count than cDC and the CD8^+^ and CD8^−^ cDC subsets after BCG infection. However, the expression levels of CD40, CD80, CD86, and MHC-II molecules were significantly upregulated in splenic cDC and the CD8 cDC subsets compared to pDC during BCG infection. Splenic cDC had a higher expression of IFN-γ and IL-12p70 than pDC, whereas pDC had higher levels of TNF-α and MCP-1 than cDC in mice infected with BCG. At early stages of immunization with BCG containing the Ag85A protein, splenic cDC and pDC could present the Ag85A peptide to a specific T hybridoma; however, cDC had a stronger antigen presenting activity than pDC. In summary, splenic cDC and pDC extensively participate in mouse immune responses against BCG infection in vivo. Although pDC had a higher BCG uptake, cDC induced stronger immunological effects, including activation and maturation, cytokine production, and antigen presentation.

## 1. Introduction

Tuberculosis (TB) is a zoonosis characterized by chronic respiratory infections and is mainly caused by *Mycobacterium tuberculosis* (MTB). TB is an ancient disease recorded 5000 years ago. MTB was first identified as the causative agent by Robert Koch [1]. Currently, an estimated 8 million new TB cases and 1.3 million deaths per year are reported worldwide [2]. TB remains the leading cause of human death by any single infectious agent and is responsible for one of the heaviest disease burdens globally.

MTB is an intracellular bacterium that causes chronic infections. Most individuals infected with this bacterium exhibit latent TB infection [3,4]. The host immune response to the pathogen determines the occurrence and development of the disease. Based on previous studies, dendritic cells (DCs), the most powerful professional antigen presenting cells (APC), play a key role and act as a bridge between natural and acquired immune responses against mycobacterial infection in vitro and in vivo by presenting antigens to circulating naive and memory T cells and initiating immune responses [5,6,7,8,9,10]. However, DCs are a highly heterogeneous population composed of many subsets characterized by different phenotypes and functions. Conventional DC (cDC) and plasmacytoid DC (pDC) are the main DC subsets in the mouse spleen during steady state. The cDC subset can be further divided into CD8^+^ and CD8^−^ cDC based on the expression of CD8α [11,12,13]. A subset of pDC displays a plasma morphology and is termed Type 1 interferon-producing cell (IPC) as it rapidly secretes massive amounts of Type 1 interferon [14]. Recent accumulated evidence indicates that tumor-infiltrating pDCs promote recruitment of regulatory T cells into the tumor microenvironment, leading to immunosuppression and promoting tumor growth [15]. By performing evaluations on the specific immunological functions of different DC subsets in response to TB, our understanding of the immunological process of TB infection will be markedly improved. However, only a few reports have been published on this subject, and little is known about the DC subsets, especially pDC, which are involved in immune responses against mycobacterial infection. Such limited knowledge is partly due to the low number of DCs in vivo and difficulties in their purification and preparation. 

Our previous study found that there are functional differentiations in activation, inflammation, and antigen presentation during BCG infection between pDC and cDC generated from mouse bone marrow progenitors stimulated with Flt3 ligand (FL-DCs) in vitro [16]. However, how the distinct characteristics of their equivalents of splenic cDC and pDC subsets in vivo need to be revealed. In the present study, the immunological characteristics, and responses of different murine DC subsets to Bacillus Calmette–Guérin (BCG) infection in vivo, including the infection rate, cellular activation and maturation, cytokine production, and specific antigen presentation ability, were evaluated to improve our understanding of the immunological functions of different DC subsets during mycobacterial infection.

## 2. Materials and Methods 

### 2.1. Mice and Microorganisms

Specific pathogen-free female C57BL/6 (H-2K^b^ and I-A^b^) mice (age, 6–8 weeks old) were purchased from Beijing Vital River Laboratory Animal Technology Co., Ltd. (Beijing, China). Mice were housed under pathogen-free, environmentally controlled conditions in the animal facility at our institute. The animals were maintained and handled with care, and all experiments were approved by the Animal Care and Use Committee of Yangzhou University with approval Number of SJXY-8.

The *M. bovis* BCG Pasteur vaccine strain 1173P2 was kindly provided by Dr. Kanglin Wan (Chinese Center for Disease Control and Prevention, Beijing, China), and green fluorescent protein expressed recombinant BCG (rBCG-GFP) was donated by Dr. Xiaoming Zhang (Institut Pasteur of Shanghai, Chinese Academy of Sciences, Shanghai, China). These strains were grown as dispersed bacilli with gentle agitation in Middlebrook 7H9 fluid medium (BD Difco, Detroit, MI, USA) supplemented with 0.05% Tween 80 and 10% Middlebrook ADC Enrichment (BD Difco) for approximately 14 d. The strains were divided into small aliquots and stored at −80 °C until use. The number of colony forming units (CFUs) for each bacterium was determined by plating suitable dilutions in phosphate-buffered saline (PBS) on plates of Middlebrook 7H10 agar medium (BD Difco) supplemented with 0.05% Tween 80 and 10% Middlebrook OADC Enrichment (BD Difco). Bacteria were grown for approximately 4 weeks for colony counting using the naked eye. 

### 2.2. T Cell Hybridoma and Antigen

The MHC class II-restricted T cell hybridoma DE10 specific for the immunodominant p241–260 peptide epitope of mycobacterial Ag85A protein was kindly provided by Dr. Claude Leclerc (Institut Pasteur, Paris, France) [17]. The Ag85A protein was expressed and purified in our laboratory, as described previously [18]. 

### 2.3. Antibodies for Flow Cytometric Analysis

All Abs for flow cytometric analysis, including purified, fluorescein- or biotin-conjugated primary Abs and streptavidin-conjugated secondary Abs, were purchased from BD Pharmingen (San Diego, CA, USA), unless otherwise stated. Flow cytometric analysis was performed using a FACS Calibur with Cell Quest Pro software (BD, San Jose, CA, USA), and the data were analyzed using FlowJo software (Tree Star, San Carlos, CA, USA).

### 2.4. Mouse Immunization and Preparation of the Splenic cDC and pDC Subsets

Groups of C57BL/6 mice were immunized intravenously with BCG (1 × 10^8^ CFU), rBCG-GFP (1 × 10^7^ or 1 × 10^8^ CFU), and purified Ag85A (100 μg) in 100 μL PBS for 4, 12, 24, and 48 h, respectively. Another group of unvaccinated mice, which received an equal volume of PBS alone, served as the control group (also referred as 0 h group) in this study. Each group contained 8 mice based on the power analysis using GPOWER software. The spleens of mice of each group were collected into one dish, perfused with RPMI 1640 medium containing 400 U/mL collagenase type IV (Invitrogen, Carlsbad, CA, USA) and 50 U/mL DNase I (Invitrogen), minced into small pieces, and incubated at 37 °C for 20 min. The pooled single whole splenic cell (WSC) suspension of each group was prepared after the disruption of red blood cells using ACK lysis buffer (Invitrogen) and filtered through a 70-μm cell strainer (Miltenyi Biotec, Bergisch Gladbach, Germany). 

Alternatively, splenic pDC and cDC subsets were enriched with an autoMACS magnetic cell separator by staining with anti-mPDCA-1 and CD11c microbeads (Miltenyi), respectively, according to the manufacturer’s instructions. A two-step method was designed to purified cDC and pDC: pDCs were firstly purified using the WSC suspension, and then cDCs were purified from the negative fraction after pDC purification. The purity of the isolated cDC and pDC was evaluated using FACS Calibur after staining with purified anti-mouse CD16/CD32 to block nonspecific binding, and then FITC-labeled anti-CD11c and PE-labeled anti-CD45RA mAbs were washed twice with FACS buffer (PBS containing 0.1% bovine serum albumin [BSA] and 0.1% sodium azide). 

### 2.5. Infection Rate Analysis of Differential Splenic DC Subsets

Pooled spleens were obtained from mice intravenously injected with PBS (0 h) and 1 × 10^8^ rBCG-GFP for 4, 12, 24, and 48 h, and the WSCs were prepared as described in Section 2.4 of this study. Approximately 1 × 10^6^ WSCs from each group (*n* = 8) were aliquoted into tubes and stained with purified CD16/CD32 and surface markers, including biotinylated anti-CD11c, PE-labeled anti-CD45RA, and APC-labeled CD8α MAbs in FACS buffer. Cells were then washed twice, incubated with PerCP-conjugated streptavidin, and washed again before analysis using FACS Calibur to determine the infection rate of differential splenic DC subsets.

### 2.6. CFU Counting of Intracellular Bacteria in Splenic cDC and pDC

Splenic cDC and pDC were obtained from mice injected with 10^8^ rBCG-GFP for 4, 12, 24, and 48 h as described in Section 2.4 of this study. A total of 1 × 10^6^ cells from each group (*n* = 8) was lysed in PBS plus 0.1% Triton X-100 (Sigma, St. Louis, MO, USA) for 10 min, and then 10-fold dilutions of cell lysates in PBS were cultured on 7H10 plates as described in Section 2.1 of this study. The CFU of intracellular bacteria was counted by eye after the plates were incubated at 37 °C for approximately 4 weeks.

### 2.7. Analysis of the Expression of Surface Molecules on Differential Splenic DC Subsets

Pools spleens were obtained from mice injected with PBS (0 h) and 10^8^ BCG for 4, 12, 24, and 48 h, and the WSC was prepared as described in Section 2.4 of this study. Approximately 5 × 10^6^ WSC from each group (*n* = 8) was divided into five aliquots and stained with purified CD16/CD32 surface markers, including FITC-labeled anti-CD11c, PE-labeled anti-CD45RA, APC-labeled CD8α, and biotinylated anti-CD40, CD80, CD86, and I-Ad (MHC-II) MAbs in FACS buffer. Cells were then washed twice, incubated with PerCP-conjugated streptavidin, and washed again before analysis using FACS Calibur to evaluate the expression of these molecules in differential splenic DC subsets.

### 2.8. Measurement of Cytokine Production by Splenic cDC and pDC

Splenic cDC and pDC were prepared from mice injected with PBS (0 h) and 10^8^ BCG for 4, 12, 24, and 48 h as described in Section 2.4 of this study. The cells of each group (*n* = 8) were washed twice with complete RPMI 1640 medium (CM) supplemented with 10% fetal bovine serum, 100 μg/mL streptomycin, 100 U/mL penicillin, and 50 μM mercaptoethanol (Invitrogen), and then added to 96-well microplates in 200 μL CM containing 1 × 10^6^ cells per well. The supernatant from each well was harvested after incubation at 37 °C with 5% CO_2_ for 24 h, incubated with Cytometric Bead Array (CBA) Mouse Inflammation Kit (BD Biosciences), and detected using FACS Calibur according to the manufacturer’s instructions. The concentrations of TNF-α, IL-6, IL-10, IL-12p70, and MCP-1 in the samples were calculated using standard curves and expressed as pg/mL.

### 2.9. Ex Vivo Antigen Presentation Assay for Splenic cDC and pDC

Splenic cDC and pDC were obtained from mice injected with PBS (0 h) and the Ag85A protein for 4, 12, and 48 h as described in Section 2.4 of this study. The cells of each group (*n* = 8) were washed twice with CM, added to 96-well microplates, and serially diluted with CM. A total of 1 × 10^5^ T cell hybridoma (DE10) cells which specifically recognize Ag85A were added to these wells and incubated at 37 °C with 5% CO_2_ for 24 h. The supernatants from each well were harvested and assayed in duplicate for IL-2 content using a Sandwich ELISA kit (BD Pharmingen) according to the manufacturer’s instructions. 

### 2.10. Statistical Analysis

All experiments were repeated at least three times with consistent results, and statistical comparison of the results of cDC and pDC at one time point was performed using an unpaired Student’s *t*-test using Prism 6 software (GraphPad Inc., San Diego, CA, USA). Values are expressed as mean ± SD, and *p* < 0.05 was considered to indicate significant difference (*), while *p* < 0.01 was considered to indicate very significant difference (**).

## 3. Results

### 3.1. Cell Sorting of Murine Splenic cDC and pDC

**Figure 1:**
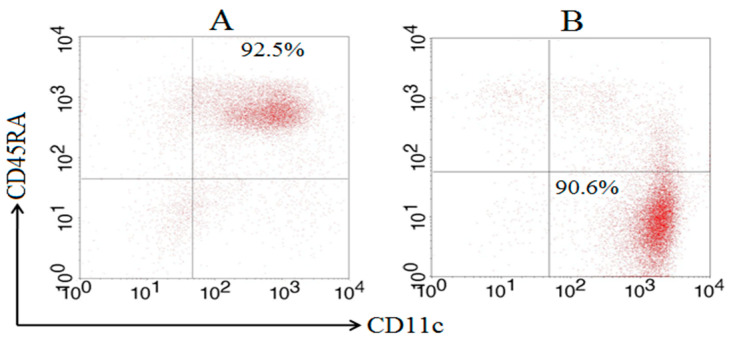
Purity of splenic cDCs and pDCs after MACS sorting based on FACS. Splenic pDC was prepared using a WSC suspension, and splenic cDC was subsequently prepared using the negative fraction of the pDC purification by MACS sorting after staining with anti-mPDCA-1 and CD11c microbeads, respectively. The purity of the pDC (**A**) and cDC (**B**) subsets was determined using FACS Calibur after staining with FITC-labeled anti-CD11c and PE-labeled anti-CD45RA MAbs.

Previous studies found that murine splenic total DCs are primarily responses to mycobacteria in the early time after infection [7,19]. So, time points of 4 h, 12 h, 24 h, 48 h were chosen to further analyze the immune responses of cDCs and pDCs subsets after BCG infection in this study. Splenic pDCs were prepared using the WSC suspension collected from C57BL/6 mice with a purity of 92.5% (Figure 1A). Splenic cDCs with a purity of 90.6% were prepared using the negative fraction after pDC purification (Figure 1B). AutoMACS sorting of murine subcutaneous cDC and pDC was repeated in different experiments with a similar purity, as described here.

### 3.2. In Vivo Infection of Different DC Subsets

**Figure 2:**
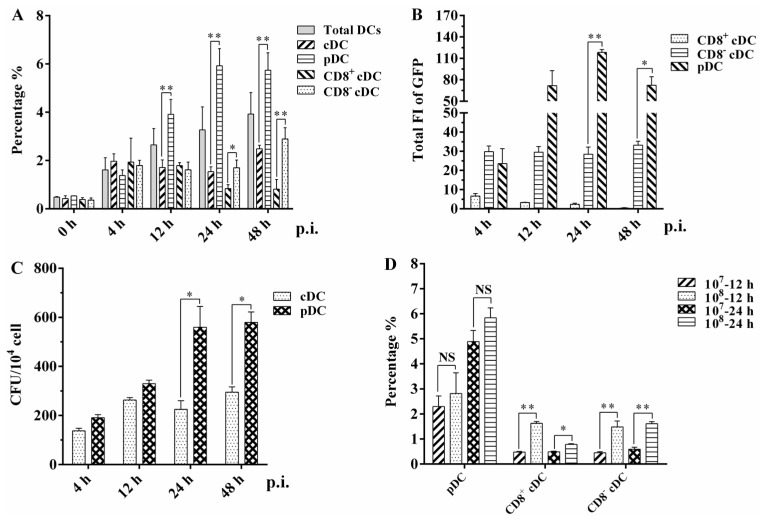
Infection analysis of different splenic DC subsets in mice injected with rBCG-GFP: (**A**) Infection rate of murine splenic total DC, cDC, pDC, CD8^+^ cDC, and CD8^−^ cDC was analyzed using FACS at different hours post-infection (p.i.). (**B**) Total GFP fluorescence intensity (FI) of murine splenic CD8^+^ cDC, CD8^−^ cDC, and pDC was calculated by the mean GFP FI multiplied the cell number of each DC subset among 10^4^ splenic cells. (**C**) CFU counting of intracellular rBCG-GFP in splenic cDC and pDC subsets; (**D**) infection rate of splenic CD8^+^ cDC, CD8^−^ cDC, and pDC subsets when mice were immunized with different CFUs of rBCG-GFP. The experiment was repeated three times, and data are shown with the mean value of the three independent experiments. The bar graphs show the mean ± SD. * *p* < 0.05; ** *p* < 0.01; NS, no significance.

The recombinant green fluorescent protein (GFP) expressing the BCG Pasteur strain (rBCG-GFP) was used to examine the internalization ability of different murine DC subsets, and the infection rates (GFP-positive rate) of total DCs, cDC, pDC, and the CD8^+^ cDC and CD8^−^ cDC subsets of the spleen were determined using flow cytometry (FACS) following intravenous (i.v.) administration of rBCG-GFP to mice (Figure 2A). After immunization, the infection rate of the total DCs increased slightly from 1.61% to 3.92%. The respective infection rate of different DC subsets was 1.97% ± 0.31, 1.71% ± 0.31, 1.54% ± 0.20, and 2.48% ± 0.15 for cDC, and 1.38% ± 0.23, 3.91% ± 0.62, 5.92% ± 0.71, and 5.74% ± 0.72 for pDC at 4, 12, 24, and 48 h post-infection. The CD8^+^ cDC, CD8^−^ cDC, and total cDC subsets displayed a similar infection rate at 4 h and 12 h. However, the infection rate of CD8^+^ cDC decreased to approximately 0.8%, while that of CD8^−^ cDC remained stable or increased at later infection time points. A significantly higher infection rate was obtained with pDC than cDC at 12 h (*p* = 0.0053), 24 h (*p* = 0.0005), and 48 h (*p* = 0.0015), and CD8^−^ cDC than CD8^+^ cDC at 24 h (*p* = 0.013) and 48 h (*p* = 0.0041) post-infection.

The total GFP fluorescence intensity (FI) of splenic CD8^+^ cDC, CD8^−^ cDC, and pDC among 10^4^ splenic cells (mean FI [MFI] of GFP × cell number of each DC subset) was calculated to evaluate the bacterial load of BCG in different mouse splenic DC subsets (Figure 2B). CD8^+^ cDC had a significantly lower total GFP FI than CD8^−^ cDC and pDC at all time points post-infection. The total GFP FI of CD8^−^ cDC was also significantly lower than that of pDC at 24 h (*p* = 0.0019) and 48 h (*p* = 0.045). These clear differences in total GFP FI imply a variable intracellular bacterial load among the different splenic DC subsets of mice immunized with BCG. CFU counting was used to determine the number of intracellular rBCG-GFP surviving splenic cDC and pDC subsets following mouse immunization and autoMACS purification (Figure 2C). Compared with splenic cDC, the number of bacteria in splenic pDC increased as the immunization time extended and was significantly higher than that in cDC at 24 h (*p* = 0.035) and 48 h (*p* = 0.013). These results and the infection rate observations obtained using FACS highlight the capacity of both murine splenic cDC and pDC to internalize bacilli during BCG infection in vivo.

Different CFUs of rBCG-GFP were intravenously injected into mice to further assess the kinetics of the infection rate of differential splenic DC subsets (Figure 2D). Interestingly, the infection rate of pDC did not differ significantly between mice immunized with 10^7^ or 10^8^ bacilli at 12 h (*p* = 0.515) and 24 h (*p* = 0.150) post-infection. However, both CD8^+^ cDC and CD8^−^ cDC had a significantly lower infection rate in mice immunized with 10^7^ rBCG-GFP than in those immunized with 10^8^ bacilli at 12 h (*p* = 0.0025 and 0.025, respectively) and 24 h (*p* = 0.0078 and 0.0063, respectively). These results suggest that splenic pDC has a higher and more stable infection rate than cDC, indicating that pDC might be more susceptible to BCG infection than other mouse DC subsets.

### 3.3. Expression of Surface Molecules on Different Splenic DC Subsets

**Figure 3:**
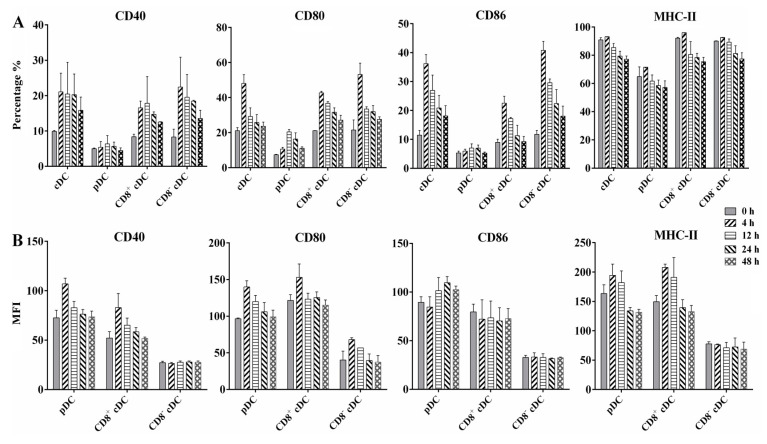
Flow cytometric analysis of surface costimulatory and MHC-II molecules expressed on differential murine splenic DC subsets. WSCs were obtained from naive mice (0 h) or 10^8^ BCG injected mice at different time points, and stained with a panel of MAbs to detect CD40, CD80, CD86, and MHC-II on splenic cDC, pDC, CD8^+^ cDC, and CD8^−^ cDC. (**A**) Percentage of positive cells is indicated for each panel; (**B**) mean fluorescence intensity (MFI) of surface molecules expressed on different DC subsets; the experiment was repeated four times, and data are shown with the mean value of the four independent experiments. The bar graphs show the mean ± SD.

The results of infection rate and intracellular bacteria counting revealed the ability of different mouse splenic DC subsets to internalize BCG bacilli during in vivo infection. To determine whether BCG infection induced the maturation of DC subsets, we analyzed the cell surface marker expression of splenic cDC, pDC, CD8^+^ cDC, and CD8^−^ cDC from naive or BCG-immunized mice, including the costimulatory molecules CD40, CD80, and CD86, and MHC class II (MHC-II) molecules, using FACS. An increase in the percentage of surface molecule-positive cells was observed for each DC subset infected with BCG compared with the PBS control (Figure 3A). In particular, splenic cDC expressed higher levels of CD40, CD80, and CD86 from 4 h to 48 h post-infection compared with 0 h, especially at 4 h, with values of 21.1% ± 5.3 (*p* = 0.028), 48.1% ± 5.0 (*p* = 0.018), and 36.2% ± 3.1 (*p* = 0.009), respectively. A similar expression of CD40, CD80, CD86, and MHC-II was observed for CD8^+^ cDC and CD8^−^ cDC, aligning with the results of cDC. In contrast, although these four molecules were upregulated in splenic pDC during BCG infection, the percentage of positive cells for each molecule was markedly lower than that of cDC subsets and was not statistically significant, except for the CD80 molecule at 12 h (20.5% ± 1.4, *p* = 0.005), compared with naive pDC. 

The MFI of each molecule was calculated to evaluate their expression intensity on the corresponding molecule positive cells (Figure 3B). Interestingly, pDC showed increased MFI for CD40, CD80, CD86, and MHC-II on the molecules positive pDC, respectively, after BCG immunization, especially at 4 h and 12 h post-infection, suggesting high expression levels of these molecules on the limited positive pDC. CD8^+^ cDC displayed MFI changes for CD40, CD80, and MHC-II-like pDC, whereas CD8^−^ cDC displayed similar MFI for each molecule as the PBS control at different time points. These results indicate that differential splenic DC subsets undergo functional activation and maturation during the early stages of BCG infection, although variable expression of active markers exists among DC subsets.

### 3.4. Cytokines Production by Splenic cDC and pDC

**Figure 4:**
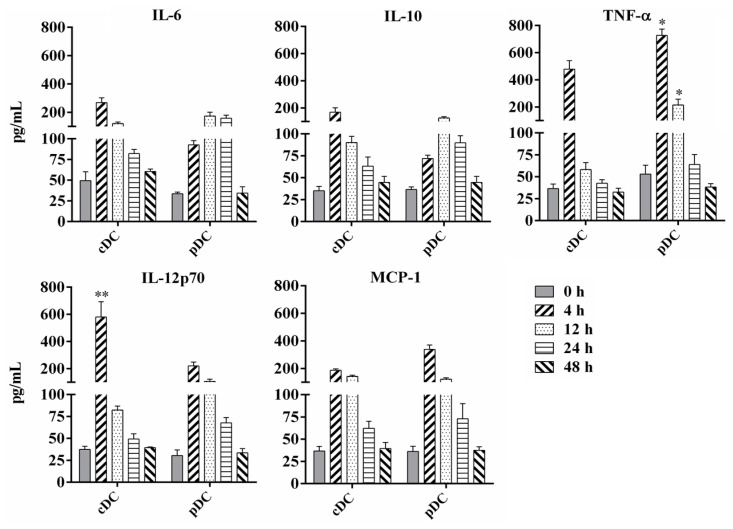
Analysis of cytokine production by murine splenic cDC and pDC following BCG infection. Splenic cDCs and pDCs were collected from naive mice or 10^8^ BCG injected mice at different time points and cultured at 37 °C with 5% CO_2_ for 24 h. Cytokine concentrations of TNF-α, IL-6, IL-10, IL-12p70, and MCP-1 in the cell culture supernatant were analyzed using FACS Calibur after staining with Cytometric Bead Array (CBA) Mouse Inflammation Kit. The experiment was repeated three times, and data are shown with the mean value of the three independent experiments. The bar graphs show the mean ± SD. * *p* < 0.05; ** *p* < 0.01.

DC-derived cytokines play a critical role in T cell priming and differentiation and the mediation of inflammatory responses. We analyzed the profile and kinetics of inflammatory cytokines secreted by murine splenic cDC and pDC after BCG infection at different time points. As shown in Figure 4, BCG infection significantly stimulated both splenic cDC and pDC to produce IL-6, IL-10, IL-12p70, MCP-1, and TNF-α, especially at 4 h and 12 h post-infection, whereas naive DCs secreted negligible amounts of these cytokines. The production of IL-12 from cDC was significantly higher than that from pDC at 4 h post-infection (*p* = 0.0003), whereas the secretion of TNF-α from pDC was significantly higher than that from cDC at 4 and 12 h post-infection (*p* = 0.045 and 0.034, respectively). These results indicate that splenic cDC and pDC may play different roles in the regulation of innate immune and primary T cell responses during the early stages of BCG infection in mice.

### 3.5. Antigen Presentation by Splenic cDC and pDC

**Figure 5:**
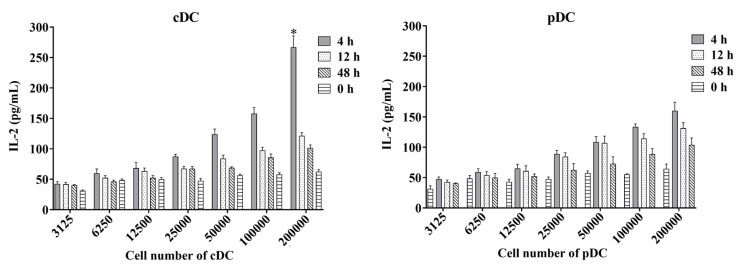
Antigen-presenting activity of mouse splenic cDC and pDC measured using the ex vivo assay. Murine splenic cDCs and pDCs from mice immunized with the mycobacteria-derived Ag85A protein at different time points were used to directly stimulate DE10, a T cell hybridoma, at 37 °C with 5% CO_2_ for 24 h. The amount of IL-2 in culture supernatants was determined using sandwich ELISA and a standard curve generated from recombinant IL-2. The experiment was repeated two times and data are shown with the mean value of the two independent experiments. * *p* < 0.05.

We proceeded to assess the kinetics of the antigen-presenting activity of splenic cDC and pDC subsets by immunizing mice with a mycobacteria-derived Ag85A protein for 4, 12, and 48 h. The antigen presentation of Ag85A by splenic cDC and pDC were detected ex vivo by measuring the amount of IL-2 in the supernatants of DCs co-cultured with DE10 (Figure 5). Both splenic cDC and pDC were found to have high antigen-presenting activity at 4 and 12 h after immunization, but not at 48 h. This decline over time implied that the highest level of Ag85A peptide presentation by splenic cDC and pDC may occur at 4 h or even earlier after immunization. Meanwhile, splenic cDC with cell number of 200,000 produced significantly higher levels of IL-2 than that of splenic pDC at 4 h (*p* = 0.022), indicating a stronger capacity for antigen-presenting activity during BCG infection in mice.

## 4. Discussion

MTB is an intracellular pathogen whose infection is mainly controlled by the immune responses induced by T helper 1 (Th1) CD4^+^ T cells secreting cytokines, such as IFN-γ [20,21]. This anti-mycobacterial protective immunity is well known to be triggered and maintained by DCs, depending on the ability of cytokines production and antigen presentation. Although few previous studies have revealed how different DC subsets respond to mycobacterial challenges, these studies were limited to a small range of DC subsets [22,23]. To further improve our understanding of the role of different DC subsets in mycobacterial infection, we evaluated the early phase immune events of splenic DC subsets in mice infected with BCG.

Although macrophages (MQ) might be the principal host cells in which MTB resides during infection, recent studies have verified the presence of bacteria in DCs of both mice and humans [6,24]. In this study, we used GFP-labelled BCG to further analyze its distribution in the splenic cDC and pDC subsets of mice after intravenous infection. Approximately 2% to 4% of GFP-positive total DCs in the mouse spleen were detected, which was significantly lower than that in granulocyte-macrophage colony stimulating factor (GM) stimulated DCs (GM-DCs) in vitro [5,25]. These obviously different infection rates revealed the difference in phagocytic ability between these two categories of DCs. Thus, GM-DCs might not reflect the true immune functions of DCs in vivo. Interestingly, the FL-DCs showed much nearer infection rates with splenic DCs than GM-DCs, indicating that FL-DCs act as closer equivalents of steady-state splenic DC subsets [16,26]. Another obvious difference observed was the higher infection rate of splenic pDC than that of cDC based on the intracellular live BCG counting and total GFP FI results, suggesting that murine splenic pDCs have a stronger phagocytic ability to internalize MTB bacilli than splenic cDC. Moreover, a similar infection rate was observed for splenic pDC, whereas the rate for both CD8^+^ and C8^−^ cDC was significantly decreased by infection with a lower dose of BCG, further indicating that pDC might be more susceptible to BCG infection than other DC subsets in vivo. Previous studies found that unlike MQ, human DCs are not permissive for the growth of mycobacteria [27,28]. In this study, the amount of BCG in total DCs seems to be stable, which was mainly reflected in cDC rather than pDC. The different receptors utilized for antigen entry may influence the capacity of cDC and pDC to internalize BCG. In addition, the maturation status determines the distinct phenotypic and functional modalities of DCs. For example, immature DCs (iDCs) are programmed for antigen capture and highly express a range of endocytic and phagocytic receptors, whereas mature DCs (mDCs) are poor at capturing antigens as most receptors associated with antigen uptake are downregulated [29]. Our study showed no significant difference in infection rate between cDC and pDC at an early time point (4 h), indicating that splenic pDC may need more time for maturation than cDC.

After antigen uptake, iDCs undergo maturation characterized by the upregulation of cell surface receptors, including costimulatory molecules, such as CD80, CD86, CD40, CD54, MHC-I, and MHC-II [30,31]. In this study, the expression of CD40, CD80, CD86, and MHC-II molecules in different splenic DC subsets was detected in mice immunized with BCG using FACS. CD40, CD80, CD86, and MHC-II were upregulated in both cDC and its CD8^+^ and CD8^−^ cDC subsets, indicating the efficient activation and maturation of cDC in response to BCG infection. However, the surface density of these molecules was only increased in CD8^+^ cDC, suggesting their greater activation than in CD8^−^ cDC. However, DCs in active pulmonary tuberculosis patients expressed lower levels of HLA-DR and CD80 than those from healthy controls [32,33]. These different expression profiles of the molecules may be regulated by the difference of subsets and location of DCs between humans and mice. Interestingly, the positive rate of CD40, CD80, CD86, and MHC-II were only slightly increased in splenic pDC and were significantly lower than those in cDC, indicating less efficient activation and maturation for the whole pDC population during BCG infection. The similar increase in the MFI values of these molecules in pDC and CD8^+^ cDC implied that splenic pDC can also undergo efficient activation and maturation during BCG infection. However, this activation and maturation of pDC was strictly limited to the very small proportion of pDC with positive surface molecule expression. Previous studies and our data implied that only a few DCs can phagocytize mycobacteria, but the upregulation of the surface molecules usually occurs in a large population of DCs, indicating that direct interaction and bystander effects mediate DC activation and maturation during mycobacterial infection [6,24,34,35]. However, our study implied that this extensive activation and maturation of DCs might only occur in cDC, whereas only pDC that directly encounter bacilli were activated to mature.

The cytokine production of MTB-activated DCs determines subsequently efficient and effective interactions with T cells. We measured the amounts of inflammatory cytokines secreted in the cell culture supernatants of splenic cDC and pDC from mice immunized with BCG. Compared to naive control mice, a similar increase in the secretion of IL-6, TNF-α, MCP-1, and IL-12 was observed in splenic cDC and pDC of BCG-infected mice. All cytokines reached a peak level at 4 h, indicating the strongest cytokine production activity of these DCs at an early time point post BCG infection, accompanied by the activation and maturation of DCs. IL-6 is a key proinflammatory cytokine during acute MTB infection that promotes Th17 differentiation and inhibits regulatory T cell differentiation [36,37]. TNF-α also plays a central role in the initiation and maintenance of the immune response to mycobacterial infections. TNF-α is essential for cell migration, granuloma formation, and clearance of bacilli from mice infected with MTB [38,39] and plays a key role in maintaining latent TB infections in the clinic [40,41]. Another important cytokine that controls mycobacterial infection is IL-12, which is critical for DC migration, antigen presentation, and promotion of IFN-γ secretion by CD4^+^ T cells [42]. The susceptibility of IL-12p40- or IL-12 receptor-deficient mice to MTB strongly supports its important role in the protective immune response against TB [43,44]. Previous evidence has been reported to suggest that pDC as well as cDC play roles in mycobacteria infection including by cooperation with CD1c^+^ DC to promote the stimulation of CD4^+^ T cells of TB patients [45,46]. This study found a similar increase in the production of these cytokines by both cDC and pDC, indicating that splenic cDC and pDC can play important roles in protective antimycobacterial immune responses by cytokines production. Moreover, splenic cDC secreted significantly higher levels of IL-12p70 than pDC, indicating the role of cDC in antigen presentation and IFN-γ secretion to promote Th1 immunity. However, splenic pDC secreted more TNF-α and MCP-1 than cDC, indicating a stronger capacity to produce inflammatory cytokines during BCG infection in mice. 

Another key role of DCs is to present antigens to circulating naïve and memory T cells to initiate cellular immune responses against mycobacteria. Although both MQ and DCs can process and present mycobacterial antigens to T cells, several recent studies revealed that DCs are the primary APC for naive CD4^+^ T cells and CD8^+^ T cells [6,21,47,48]. In this study, we further investigated the antigen-presenting activity of splenic cDC and pDC using an MHC-II-restricted T hybridoma cell line specific for Ag85A protein. High levels of IL-2 were detected in both splenic cDC and pDC culture supernatants, suggesting that both of them can present Ag85A epitope in vivo. Furthermore, dynamic analysis revealed that the efficient antigen-presenting activity of both DC subsets is an early event with a peak at 4 h post-infection or even earlier, which corresponds with previous findings [6,7]. Splenic cDC exhibited a stronger capacity for antigen presentation, indicating that cDC may play a more important role in presenting antigens and initiating T cell immune responses than pDC during mycobacterial infection. 

## 5. Conclusions

In summary, we demonstrated that different splenic DC subsets, cDC and pDC, can extensively participate in immunity against *M. bovis* BCG in mice. Although splenic pDCs display a greater capacity to internalize bacilli and induce an inflammatory response, splenic cDCs play important roles in activation and maturation, and antigen presentation to trigger T cell immune responses in vivo. This discrepancy of cDC and pDC in response to mycobacteria infection will provide more precise potential targets for prevention and control of TB including vaccine design and disease progression monitoring and so on.

## Data Availability

Not applicable.
